# Deep Reconstruction Transfer Convolutional Neural Network for Rolling Bearing Fault Diagnosis

**DOI:** 10.3390/s24072079

**Published:** 2024-03-24

**Authors:** Ziwei Feng, Qingbin Tong, Xuedong Jiang, Feiyu Lu, Xin Du, Jianjun Xu, Jingyi Huo

**Affiliations:** School of Electrical Engineering, Beijing Jiaotong University, Beijing 100044, China; 22110480@bjtu.edu.cn (Z.F.); xdjiang@bjtu.edu.cn (X.J.); 21117039@bjtu.edu.cn (F.L.); xdu@bjtu.edu.cn (X.D.); jjxu@bjtu.edu.cn (J.X.); jyhuo@bjtu.edu.cn (J.H.)

**Keywords:** intelligent fault diagnosis, transfer learning, domain adaptation, autoencoder, label smoothing

## Abstract

Deep transfer learning has been widely used to improve the versatility of models. In the problem of cross-domain fault diagnosis in rolling bearings, most models require that the given data have a similar distribution, which limits the diagnostic effect and generalization of the model. This paper proposes a deep reconstruction transfer convolutional neural network (DRTCNN), which satisfies the domain adaptability of the model under cross-domain conditions. Firstly, the model uses a deep reconstruction convolutional automatic encoder for feature extraction and data reconstruction. Through sharing parameters and unsupervised training, the structural information of target domain samples is effectively used to extract domain-invariant features. Secondly, a new subdomain alignment loss function is introduced to align the subdomain distribution of the source domain and the target domain, which can improve the classification accuracy by reducing the intra-class distance and increasing the inter-class distance. In addition, a label smoothing algorithm considering the credibility of the sample is introduced to train the model classifier to avoid the impact of wrong labels on the training process. Three datasets are used to verify the versatility of the model, and the results show that the model has a high accuracy and stability.

## 1. Introduction

As one of the most important parts of many mechanical equipment, a rolling bearing is prone to failure due to a poor working environment, long working cycle, and other reasons. Faulty bearings may damage mechanical equipment, leading to catastrophic accidents [1]. Therefore, finding an intelligent and efficient bearing fault diagnosis method can improve safety in the process of mechanical use and reduce the occurrence of accidents. In recent years, fault diagnosis based on deep learning has become a hot research field and has achieved impressive success in the field of computer vision [2]. Compared with the traditional fault diagnosis methods, the application of intelligent feature extraction and pattern recognition technology represented by deep learning to fault diagnosis can directly get rid of the limitations of manual data processing and prior knowledge [3,4,5], and has a higher diagnosis efficiency. Compared with the shallow machine learning method, it can extract more abundant hidden features from the original vibration signals, and has better diagnostic results and a higher accuracy [6]. A convolutional neural network (CNN) is one of the most commonly used network structures because of its excellent feature extraction ability from its weight sharing and global pooling structures. For example, Abdeljaber et al. [7] took 1D vibration signals as input data and integrated feature extraction and classification into a learning body through CNN to detect structural damage in real time. Chen et al. [8] proposed an automatic feature learning neural network, which uses two convolutional neural networks with different core sizes to automatically extract signal features of different frequencies from the original data, which has a better performance in noisy environments. Ozcan et al. [9] proposed a method to enhance bearing fault detection, which is realized by a multi-channel and multi-stage one-dimensional CNN classifier. The deep confidence network (DBN) adopts a fully connected structure, which can extract advanced features from a large number of time domain or frequency domain signals in an end-to-end form. For example, researchers such as He et al. [10,11,12] have adopted the fault diagnosis method for gear transmission chains based on the depth confidence network. Compared with traditional methods, this method can adaptively utilize the robust features related to faults, so it can rely less on signal processing technology and prior diagnostic knowledge.

Both CNNs and DBNs are commonly used monitoring algorithms, and the training process of the diagnosis model requires a large number of fault data and corresponding fault labels. In contrast, an automatic encoder (AE) is an unsupervised algorithm proposed by Hinton et al. [13], which aims to restore input data and can realize unsupervised training of the network. An AE consists of an encoder and a decoder. Compared with other methods, it has the advantages of data reduction and data reconstruction while reducing label dependence. For example, Wang et al. [14] proposed a stack-based, self-coding fault diagnosis method, which can effectively output classification results when combined with softmax regression. Sun et al.’s [15] method, based on a stacked sparse autoencoder (SAE) combined with compression sensing technology, has a better ability to identify mechanical faults. Li et al. [16] proposed a local discriminant reserved limit learning machine autoencoder (LDELM-AE) to learn the data representation of the local geometry and local discrimination in the input data. The two graphs are used to enhance the inter-class compactness and inter-class separability, respectively, and the local geometric features and discriminant features in the input data can be learned. However, the training parameters of the autoencoder are relatively few, and the fitting ability is usually weak. Therefore, on the basis of the ordinary autoencoder, the convolution autoencoder combines an AE and CNN and uses the convolutional neural network instead of the fully connected neural network. The problem of underfitting of the model can be avoided by more training parameters. The convolutional neural network in the convolution encoder can also capture the spatial structure information of the input signal more effectively.

However, the above fault diagnosis methods still have some limitations: the source domain data used to train the diagnosis model must have the same distribution as the target domain data used for testing; otherwise, it will have a low test accuracy. However, the actual operating conditions of mechanical equipment are complex and changeable, so it is difficult to meet this demand. If the diagnostic model is to be used in new working conditions, a large number of target domain fault data labels are needed. However, the real fault data are difficult to collect, and it is difficult to provide a sufficient amount of target domain-labeled data.

Deep transfer learning solves these problems well and provides a new development direction for the field of intelligent fault diagnosis. It can transfer the knowledge learned from the source domain to the diagnosis task of the target domain and improve the diagnosis effect of the model in different tasks. Guo et al. [17] proposed a deep convolutional transfer learning network (DCTLN), which includes two modules: conditional recognition and domain adaptation. Domain adaptation is realized by maximizing the domain recognition error and minimizing the probability distribution distance. The feature-based transfer neural network (FTNN) proposed by Yang et al. [18] creates a domain-shared CNN, extracts the original vibration data characteristics of the source domain and the target domain, and completes the transfer through multi-layer domain adaptation and pseudo-label learning. The above two articles use the maximum mean discrepancy (MMD) algorithm to achieve the global alignment of the source domain and the target domain. The MMD is a common algorithm for transfer learning, but it ignores the alignment of different fault subdomains. The differences between different categories can easily affect the classification results and lead to a negative transfer. Liu et al. [19] proposed a deep adversarial subdomain adaptation network (DASAN), which introduces a new difference measurement method known as the local maximum mean discrepancy (LMMD), which completes the alignment between the source subdomain and the target subdomain and avoids the influence of fault category differences. Jia et al. [20] proposed a deep normalized convolutional neural network (DNCNN) to solve the problem of uneven distribution of mechanical faults, which effectively avoids the result that the diagnosis is biased towards most health conditions and analyzes the core of the convolution layer using the NAM algorithm, which in understanding the learning mechanism of the network. Yang et al. [21] proposed a deep adversarial hybrid domain-adaptation network (DAHAN) to solve the problem of intelligent diagnosis under variable working conditions. Like many other researchers [22,23,24], the Wasserstein distance aligns the hybrid domain with the combination of homologous domain and global domain adaptation and realizes the hybrid domain adaptation. In addition to distance-based methods, some researchers [25,26,27] adopt strategies based on confrontation games to solve domain adaptation tasks. Among them, Jiao et al. [28] proposed a multi-weight domain adversarial network (MWDAN) based on confrontation to realize domain adaptation, introduced a class-level weighting mechanism to distinguish the label spaces, and used the obtained weights to correct the loss function. The knowledge transfer from a large label source domain to a small, unmarked target domain is realized. However, these methods tend to be overconfident in the training samples and do not take into account the accuracy of dataset labels. For a large number of datasets, there are likely to be a small number of wrong labels, and the network training process is highly dependent on the correct labels, which affects the accuracy of the model’s diagnosis and weakens the versatility of the model. In addition, in the process of unsupervised training, these models usually only use the labeled source domain data to train the classifier, and do not make good use of the structural information from the unlabeled original signal in the target domain.

Based on these problems, an unsupervised reconfiguration classification autoencoder model is proposed in this paper. The main contributions are as follows:

(1) A deep reconstruction transfer convolutional neural network (DRTCNN) is proposed. A DRTCNN is different from the existing unsupervised transfer learning in that it has a stronger ability to constrain the features of data and can deeply mine the structural information of fault signals and extract transferable features with better robustness. The DRTCNN consists of three parts: a five-layer convolutional encoder, a five-layer convolutional decoder, and a classifier considering tag confidence.
(2) A five-layer shared convolutional encoder is built as a part of the source domain data feature extractor and the target domain data reconstructor. The first layer uses a wide convolutional kernel with a size of 1 × 64 to effectively filter the noise in the high frequency band and capture the impact of the bearing. Using a shared coding representation to alternately learn source domain label prediction and target domain data reconstruction is helpful for the network to better extract the domain invariant features of the two domains. The signal reconstructor, constructed by a five-layer decoder and five-layer encoder, is used to reconstruct the target domain data and can fully mine the available structure information from the untagged original signal.
(3) The smooth label, cross-entropy loss assistant training classifier is introduced to consider the confidence of the sample label, reduce the impact of dataset error tags, and prevent model overfitting.
(4) A new subdomain adaptive function is introduced, which is called the local maximum mean discrepancy (LMMD) algorithm. Combined with the proposed DRTCNN, the algorithm maps the source domain and target domain data to the same potential space, minimizes the domain offset of each subdomain, and realizes the alignment between the source domain subdomain and the target domain subdomain.
(5) Through the CWRU dataset, BJTU dataset, and SEU dataset, 20 transfer experiments are carried out, and the diagnostic effect of the DRTCNN is compared with the existing transfer learning network to verify the superiority of the structure proposed in this paper. Through the ablation experiment, the effectiveness of each component of the network is verified, and the optimal selection of key dynamic tradeoff factors is discussed and analyzed.

## 2. Convolutional Autoencoder Neural Network

### 2.1. Autoencoder Structure

An automatic encoder (AE) is a kind of artificial neural network which can realize unsupervised learning. Its overall structure is composed of an encoder and decoder, and the two are symmetrical about the hidden layer. The data are first input into the encoder structure, and the encoder network can transform the high-dimensional input data into a low-dimensional code, which plays a good role in reducing the dimension. The decoder structure is a mirrored encoder structure, so it can reconstruct the input data. The optimization goal of the AE is to minimize the gap between the input original data and the output reconstructed data, which can be completed without relying on data labels, and unsupervised learning is realized.

**Figure 1:**
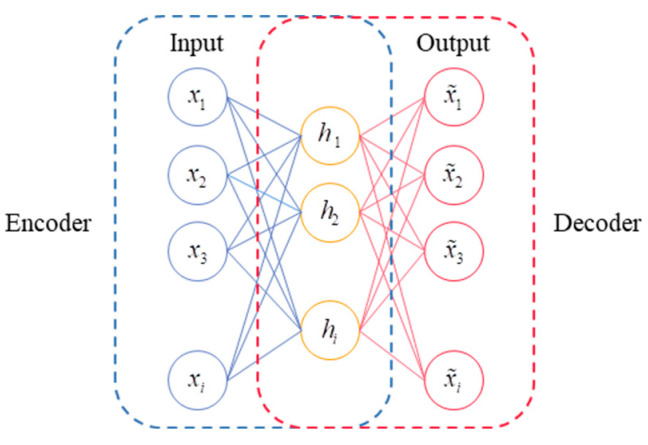
Structure of autoencoder.

The structure of an AE is shown in Figure 1. If the input sample of an AE is $X_{i}=(x_{i1},\ldots ,x_{im})$, i=1,…,n is the number of samples, j=1,…,m is the dimension of the sample, and the encoder mapping function of an AE is the sigmoid function, and it is defined as $f_{\theta }$, then the hidden layer eigenvector $h_{\theta }$ can be expressed as

(1) $$h_{\theta }^{i}=f_{\theta }\left(x^{i}\right)=S\left(Wx^{i}+b\right)$$

where *W* is the weight matrix of the coding layer and b is the offset vector of the coding layer.

Similarly, the decoder mapping function of the AE is also a sigmoid function, which is defined as the $g_{\theta }$ domain, which maps the extracted $h_{\theta }$ frames from a low-dimensional space to a high-dimensional space, thus realizing the reconstruction of the input data. For input data *x*, the corresponding refactoring data *x* values can be expressed as:

(2) $${\tilde{x}}^{i}=g_{\theta }\left(h_{\theta }^{i}\right)=\tilde{W}h_{\theta }^{i}+\tilde{b}$$

where $\tilde{W}$ is the weight matrix of the decoding layer and $\tilde{b}$ is the offset vector of the decoding layer. The parameters of the encoder and decoder will be learned at the same time to better complete the reconstruction task and strive to minimize the reconstruction error. Refactoring losses can be defined as:

(3) $$ℒ(X,\tilde{X})={\left\|X-\tilde{X}\right\|}^{2}$$

### 2.2. Design of Basic Convolutional Self-Coding Network Model

Based on the above basic structure of an autoencoder, a convolutional self-coding neural network combined with a CNN is designed to learn the characteristics of bearing data under different working conditions. Compared with the ordinary AE, a CAE uses the convolution layer instead of the full connection layer of the traditional autoencoder, and introduces the characteristics of local connection, weight sharing, and pooling of the convolutional neural network, which reduces the number of network parameters, reduces the computational complexity, and makes the model have a better generalization ability.

**Figure 2:**
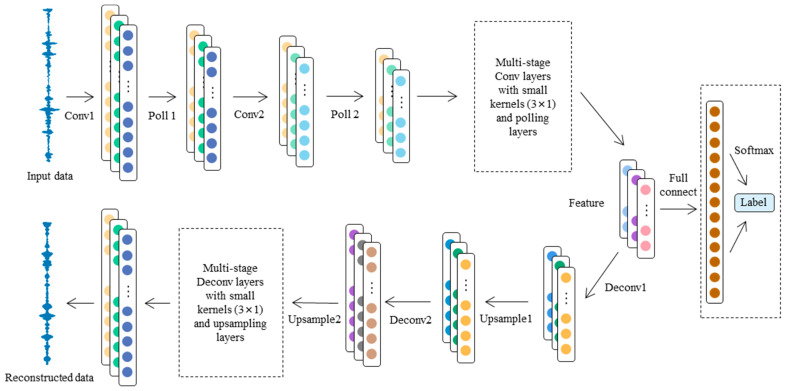
Network structure of proposed CAE.

The CAE network model feature extractor designed in this paper is the encoder of the CAE, which is made up of five one-dimensional convolution blocks to extract features from the input data. The designed network structure and parameters are shown in Figure 2. The parameters of the encoder layers and decoder layers are detailed in Table 1 and Table 2.

Each convolution block contains a convolution operation and a maximum pool operation, and is activated by the RELU function. In the first layer, we use a wide convolutional kernel of the size 1 × 64, which can effectively filter the noise in the high-frequency band and extract the effective low-frequency information in the bearing vibration signal [29], and then we use multiple small convolutional kernels with a size of 1 × 3, which can improve the time-domain resolution and enhance the nonlinear expression ability of the feature extractor by deepening the network depth. After the data are processed by the convolution block, the data are fully connected with the output layer, and the identification result of the healthy state of the bearing is output.

In the previous paper, the signal features in the target domain extracted by the encoder are input into the decoder for data reconstruction. The structure of the decoder is completely symmetrical with the encoder, which can better restore the signal and play the role of noise reduction and filtering. For the one-dimensional convolution layer in the encoder, the deconvolution layer with the same convolution core and step size is used to correspond to it; for the pooling layer in the encoder, the upper sampling layer with the same step size is used to correspond to it.

## 3. Deep Reconstructed Convolution Network

**Figure 3:**
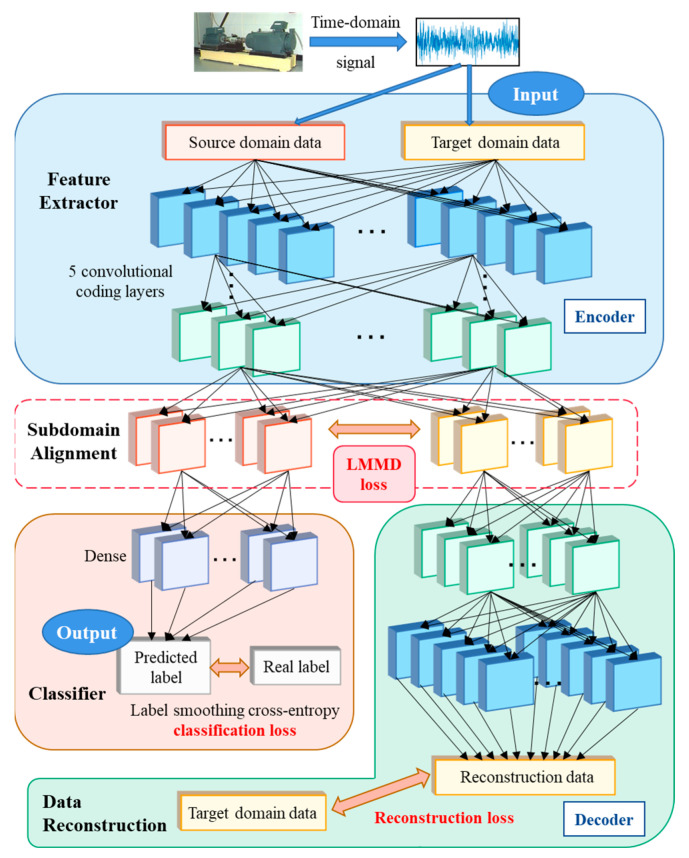
Main structure of the DRTCNN.

In this part, the optimization and training steps of the DRTCNN model will be introduced in detail. The specific structure is shown in Figure 3.

A DRTCNN is a fault diagnosis method based on an unsupervised reconfiguration transfer autoencoder, which uses the subdomain alignment algorithm as the assistant and the smooth label as the optimization. The purpose is to learn the cross-domain invariant features and finally achieve a better fault diagnosis effect across different working conditions. The network structure proposed in this paper consists of four parts: feature extraction, signal reconstruction, subdomain alignment, and classifier.

### 3.1. Unsupervised Auxiliary Training Based on Signal Reconstruction

In order to improve the universality of the diagnostic model under different working conditions, the network must have an excellent domain-invariant feature learning ability and effectively extract the common features of the source domain and target domain data, so the training of the feature extractor is very important. Our DRTCNN adopts unsupervised auxiliary training based on signal reconstruction, reconstructs the input signal through the decoder, makes full use of the structural information of the data, and shares the coding parameters at the same time, which urges the feature extractor to mine the common features of the source domain and the target domain.

The basic structure of an unsupervised refactoring classification autoencoder has been described above. The feature extraction network of the encoder is recorded as enc to extract the feature *h* from the original signals $x_{i}^{s}$ and $x_{i}^{t}$ of the source domain and the target domain. After $x_{i}^{s}$ and $x_{i}^{t}$ are input to the feature extraction layer, the encoder maps the high-dimensional sample data to the low-dimensional space to complete the extraction of the hidden layer feature *h*:

(4) $$h_{s}=enc(x_{i}^{s})$$

(5) $$h_{t}=enc(x_{i}^{t})$$

The characteristics of the target domain signal extracted by the encoder are then input into the decoder to reconstruct the unlabeled signal of the target domain. The hidden layer feature *h_t_* is mapped from the low-dimensional space to the high-dimensional data space through the decoder to obtain the reconstructed target domain data:

(6) $${\tilde{x}}_{i}^{t}=dec(h_{t})$$

where *dec* represents the network structure of the decoder. Then, the difference between the reconstructed signal and the original signal is measured using the mean square error (MSE), and the reconstruction loss is constructed:

(7) $${ℒ}_{\mathrm{rec}}(x_{i}^{t},{\tilde{x}}_{i}^{t})={\left\|x_{i}^{t}-{\tilde{x}}_{i}^{t}\right\|}^{2}=\frac{1}{N}\sum_{i=1}^{N}{\left(x_{i}^{t}-{\tilde{x}}_{i}^{t}\right)}^{2}$$

Here, *N* represents the number of samples.

The decoder reconstructs the target domain data and captures the depth characteristics of the target domain data in an unsupervised way. By minimizing the reconstruction loss to assist the feature extractor, the decoder can mine and make good use of the structural information from the target domain data, optimizing the encoder training. As a feature extractor, the training parameters of the source domain and target domain data are shared, which can effectively mine the commonness between learning tasks, provide useful information for cross-domain object recognition, and improve the domain-invariant feature learning ability of the model.

### 3.2. Domain Alignment Optimization Based on Distance

In order to improve the robustness of the model to unknown operating condition data, it is necessary to further enhance the generalization performance of the network. The source domain and target domain data are inputted into the model, and the hidden features of the source domain and target domain signals are obtained through the feature extraction of the encoder. However, due to the different distribution of the two domains, the domain offset problem easily leads to a low diagnostic accuracy. We consider introducing a distance-based domain alignment algorithm by aligning the source domain and target domain data, optimizing the new feature space by reducing the inter-class spacing, and increasing the inter-class spacing, in order to improve the learning effect of the model in the target domain.

**Figure 4:**
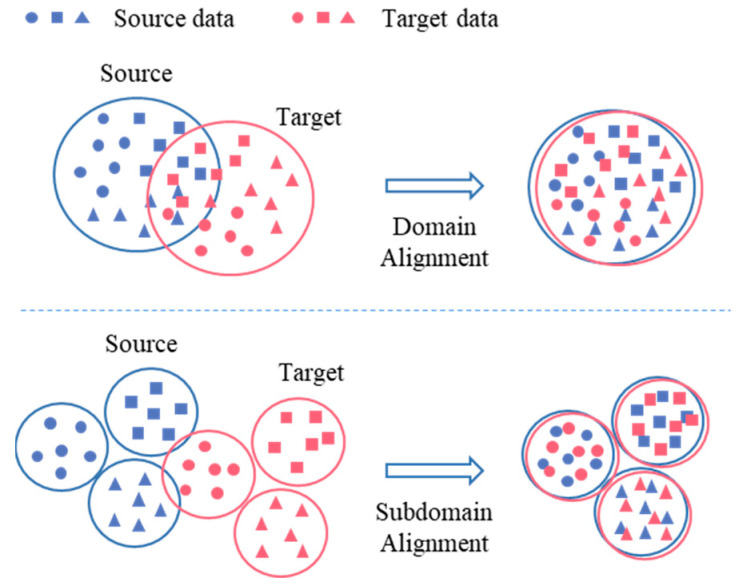
Domain alignment and subdomain alignment.

In the distance-based domain alignment algorithm, the MMD metric proposed by Gretton et al. [30] has been widely used in transfer learning [31,32,33] to minimize the difference between the source domain distribution and the target domain distribution and align the distributions of the two domains. However, this method only realizes the global alignment of the source domain and the target domain, ignores the subdomain difference based on the fault category, and loses the fine-grained message. Considering the above problems, the LMMD used in this paper can give different weights to the data with different labels, which can effectively avoid the domain offset of the subdomain [34]. On the basis of global domain alignment, we can achieve a subdomain alignment, as shown in Figure 4. In this method, the source domain $D_{s}$ and the target domain $D_{t}$ are mapped in the same reproducing kernel Hilbert space (RKHS). The expressions for measuring their differences are as follows:

(8) $$d_{ℋ}\left(p^{\left(c\right)},q^{\left(c\right)}\right)≜E_{c}{\left\|E_{p^{\left(c\right)}}\left[\phi \left(x^{s}\right)\right]-E_{q^{\left(c\right)}}\left[\phi \left(x^{t}\right)\right]\right\|}_{ℋ}^{2}$$

where *H* is the RKHS given to the characteristic kernel *k*. *φ* is a feature mapping function, which can map a given sample to the RKHS, and the characteristic kernel *k* means $k(x^{s},x^{t})=\langle \phi (x^{s}),\phi (x^{t})\rangle$, where 〈⋅,⋅〉 represents the inner product of the vector. $p^{\left(c\right)}$ and $q^{\left(c\right)}$ are the distributions of the source subdomain $D_{s}^{\left(c\right)}$ and the target subdomain $D_{t}^{\left(c\right)}$, respectively. $x^{s}$ and $x^{t}$ are the samples from the source domain and target domain, and *C* is the number of fault categories. After setting the weight $w^{c}$ for each category of samples, the unbiased estimation expression is as follows:

(9) $${\hat{d}}_{ℋ}(p^{\left(c\right)},q^{\left(c\right)})=\frac{1}{C}\sum_{c=1}^{C}{\left\|\sum_{x_{i}^{s}\in D_{s}}w_{i}^{sc}\phi \left(x_{i}^{s}\right)-\sum_{x_{j}^{t}\in D_{t}}w_{j}^{tc}\phi \left(x_{j}^{t}\right)\right\|}_{ℋ}^{2}$$

where $w_{i}^{sc}$ is the weight of sample $x_{i}^{s}$ belonging to category *c*, and $w_{j}^{tc}$ is the weight of sample $x_{j}^{t}$ belonging to category *c*. Each batch satisfies the conditions: $\sum_{i=1}^{n_{s}}w_{i}^{sc}=1$, $\sum_{j=1}^{n_{t}}w_{j}^{tc}=1$. For the input sample *x*, the corresponding *w* is calculated as follows:

(10) $$w_{i}^{c}=y_{i}^{c}/\sum_{(x_{j},\;y_{j})\in D}y_{j}^{c}$$

where $y_{i}^{c}$ is the c-th element of the vector $y_{i}$. The label of the source domain sample is the real label $y_{i}^{s}$, and the weight $w_{i}^{sc}$ of each type of sample in the source domain can be calculated by the independent heat vector *i*. However, in the unsupervised training task, the target domain sample is unlabeled and cannot be calculated directly by using Expression (9). Because the output ${\hat{y}}_{i}^{t}$ of the training network classifier is a probability distribution of the samples in the target domain, it directly represents the probability of assigning $x_{i}^{t}$ to each label *c*. Therefore, the probability of assigning $x_{i}^{t}$ to each class *c* can be well represented by ${\hat{y}}_{i}^{t}$. If the activation of the l-th feature layer is $a^{sl}$ and $a^{tl}$, and *n* is the number of given samples, then the final measurement formula for the subdomain difference is:

(11) $$\begin{array}{l} {\hat{d}}_{l}(p^{\left(c\right)},q^{\left(c\right)})=\frac{1}{C}\sum_{c=1}^{C}[\sum_{i=1}^{n_{s}}\sum_{j=1}^{n_{s}}w_{i}^{sc}w_{j}^{sc}k\left(a_{i}^{sl},a_{j}^{sl}\right)\\ \;\;\;+\sum_{i=1}^{n_{t}}\sum_{j=1}^{n_{t}}w_{i}^{tc}w_{j}^{tc}k\left(a_{i}^{tl},a_{j}^{tl}\right)\\ \;\;\;-2\sum_{i=1}^{n_{s}}\sum_{j=1}^{n_{t}}w_{i}^{sc}w_{j}^{tc}k\left(a_{i}^{sl},a_{j}^{tl}\right)] \end{array}$$

For the source domain data *x_s_* and target domain data *x_t_* participating in the training, according to the above calculation process, the loss function ${ℒ}_{\mathrm{LMMD}}$ can be set, and the loss value can be reverse broadcast to update the network parameters:

(12) $${ℒ}_{\mathrm{LMMD}}={\hat{d}}_{l}(p^{\left(c\right)},q^{\left(c\right)})$$

The learned subdomain alignment knowledge will participate in the feature extraction of the encoder and the fault classification of the classifier by the way of parameter sharing, and gradually improve the accuracy of fault diagnosis under cross-domain tasks.

### 3.3. Training Optimization Based on Label Smoothing

In order to prevent the model from being overconfident in the training samples and further improve the generalization ability of the model to the test samples, we introduce the smooth label algorithm to optimize the training process of the classifier. The cross-entropy loss of the smooth label is improved on the basis of traditional cross-entropy loss. By introducing a smoothing coefficient, it suppresses the self-confidence of the training samples, which can effectively alleviate the problem of overfitting in the training process, improve the generalization ability of models, and reduce the impact of error labels on the training results [35]. In the training processes of most deep learning networks, the output of the full connection layer is calculated by the softmax function, and all kinds of confidence values are obtained, and then the loss value is calculated using the cross-entropy formula. However, in this process, the use of hard labels has a certain risk of overfitting; in addition, if the sample has wrong labels, the model training is easily affected, which increases the training cost and reduces the posterior accuracy. Therefore, the model proposed in this paper uses smoothing labels to solve these problems.

For each input sample *x*, the probability that it belongs to each category *c* can be calculated using the following softmax formula:

(13) $$p(c|x)=\frac{\exp\left(z_{c}\right)}{\sum_{i=1}^{C}\exp\left(z_{i}\right)}$$

where *z_i_* is the unnormalized logarithmic probability. The traditional cross entropy loss function is defined as:

(14) $$l=-\sum_{c=1}^{C}q(c)\log(p(c))$$

where *q* represents the true label distribution of the sample. The core idea of the smoothing label algorithm is to introduce the smoothing parameter *ε* to reduce the self-confidence of the model, so as to reduce the possibility of overfitting. In a multi-classification problem, hard labels are mostly used; that is, the target variable is usually a unique heat vector, and the value is 1 when the classification result is correct and 0 when the result is wrong. For the input *x_i_*, its s-class probability is defined as follows:

(15) $$q\left(c|x_{i}\right)=\{\begin{array}{ll} 1 & \;\mathrm{if}\;c=c^{*}\\ 0 & \;\mathrm{otherwise}\; \end{array}$$

**Figure 5:**
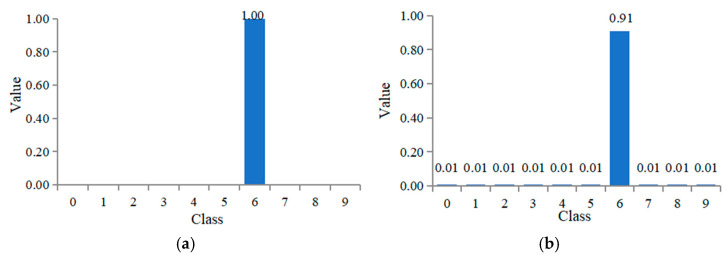
Different values for hard label and soft label. (**a**) Hard label. (**b**) Soft label.

Taking the CWRU bearing dataset as an example, 0–9 represents ten fault types in the dataset, and each type of functionality when using hard labels is shown in Figure 5a. The training process of the model will make the output probability of each sample on the correct label as 1, as possible. From the definition of the cross-entropy loss function in Expression (14), it is known that in order to attain the optimal solution, it is necessary to make the *z_i_* value close to ∞, which is easy to cause the network to overfit the training set.

If you use a soft label, that is, after introducing the smoothing parameter *ε*, the value of $q\left(s|x_{i}\right)$ is no longer non-0 or 1, but is defined as follows:

(16) $$q_{i}=\{\begin{array}{ll} 1-\varepsilon & \;\mathrm{if}\;i=y\\ \varepsilon /(c-1) & \;\mathrm{otherwise}\; \end{array}$$

where *ε* is set to a small constant, and *c* is the corresponding category. If ε = 0.09, the various possibilities corresponding to the soft label are shown in Figure 5b. Through the introduction of soft labels, the zi in the loss calculation process will no longer tend to infinity, but a specific numerical value, and the optimal solution will be in a limited range, reducing the possibility of overfitting and alleviating the impact of wrong labels. With the help of the subdomain alignment algorithm, the feature extractor can learn the domain invariant features of a given signal. Because the training classifier needs labeled data to verify the accuracy of the classification, only the extracted source domain feature *h_s_* is used as the input of the classifier. The high-dimensional features extracted by the last convolution block are first flattened through the full connection layer and transformed into one-dimensional feature vectors; the expression is as follows:

(17) $$f=a(W^{T}h^{s}+b)$$

where *f* represents the output feature vector, a(⋅) is the activation function, *h^s^* is the feature extracted by the encoder, and *W* and *b* represent the weight matrix and offset, respectively. The output one-dimensional eigenvector is then input into the softmax function, and Expression (13) finally outputs a probability vector containing the probability value of each obstacle category, in which the label corresponding to the maximum probability is the predicted fault type.

According to the predicted fault type and the real label of the source domain, the cross-entropy loss of the smooth label is calculated, that is, the classification loss of the classifier:

(18) $$\begin{array}{l} {ℒ}_{\mathrm{cls}}=-\frac{1}{N}\sum_{i=1}^{N}\sum_{c=1,c\neq c^{*}}^{C}\left(\frac{\varepsilon }{C-1}\right)\log p(c|x_{i}^{s})\\ \;\;+\left(-\frac{1}{N}\sum_{i=1}^{N}\left(1-\varepsilon )\log p(c^{*}|x_{i}^{s}\right)\right) \end{array}$$

where *N* represents the number of input samples, *C* represents the number of fault categories, *ε* represents the smooth label coefficient, which is generally set to a small constant, c* represents the real fault category, and $p(c|x_{i}^{s})$ represents the probability that the sample $x_{i}^{s}$ in the sample source domain belongs to category *c*.

### 3.4. Loss Function Considering Dynamic Trade-Off

According to the above process, the loss function used in the training model is divided into three parts: classification loss, reconstruction loss, and LMMD metric subdomain distance loss. In order to improve the convergence performance of the network and optimize the training process, we define the loss function of the network as:

(19) $$ℒ={ℒ}_{\mathrm{cls}}+\alpha {ℒ}_{\mathrm{rec}}+\beta {ℒ}_{\mathrm{LMMD}}$$

where α and β are dynamic trade-off factors. In order to obtain the best training effect, α=1 is used to ensure that the original reconstruction error is returned in the process of reconstructing the data, and the definition of β is as follows:

(20) $$\beta =-4/\left(\sqrt{\frac{e}{E+1}}+1\right)+4$$

where *e* represents the current training epoch, and *E* represents the maximum epoch value. The initial value of β is 0, and with the increase of the epoch, the effect of the LMMD loss from 0 to 1 is also enhanced with the realization of the trade-off factor, so that it can restrain noise at the beginning of training and learn basic fault characteristics better. In the later stage of training, with the increase in the β value, the learning ability of the network to the transferable features is activated and enhanced, so as to optimize the convergence of the network and obtain a better learning effect from the transferable features.

### 3.5. Method Flow of Fault Diagnosis across Working Conditions

The optimization objective of the model consists of three parts: minimizing the classification loss of the source domain, minimizing the reconstruction loss of the target domain, and minimizing the distribution distance between the source domain and the target domain. The specific training and testing process is as follows:

(1) Input data: The labeled source domain training set $D_{s}={\left\{x_{i}^{s}\right\}}_{i=1}^{n_{s}}$ and the unlabeled target domain training set $D_{t}={\left\{x_{j}^{t}\right\}}_{j=1}^{n_{t}}$ are entered, the dynamic trade-off factor *γ*, the maximum training time *E*, and the number of early stops *s* are set, and the parameter set *θ* to be trained is randomly initialized.
(2) Forward propagation: The encoder composed of five-layer convolutional blocks extracts the transferable features *h_s_* and *h_t_* of the source domain and the target domain. The prediction labels ${\hat{y}}_{s}$ and ${\hat{y}}_{t}$ of the source domain and the target domain are obtained from the full connection layer and the softmax function, the prediction labels of the source domain and the target domain are obtained, and the prediction labels of the source domain and the target domain are used to calculate the cross-entropy loss of the smooth label. The classification loss ${ℒ}_{\mathrm{cls}}$ of the samples in the source domain is obtained using Expression (18). The classification loss ${ℒ}_{\mathrm{cls}}$ is obtained by using the execution ${\hat{y}}_{t}$ in the LMMD algorithm. The distribution distance of the same label subdomain between the source domain and the target domain is calculated using Expression (12), and ${ℒ}_{\mathrm{LMMD}}$ is obtained. At the same time, the target domain signal feature ht, which is extracted by the encoder, is reconstructed by the decoder, the reconstructed signal ${\tilde{x}}_{t}$ is obtained using Expression (6), and the reconstruction loss ${ℒ}_{\mathrm{rec}}$ of the target domain data is calculated using Expression (7). When the patience of early stopping reaches 15, step (4) is performed; otherwise, step (3) is performed.
(3) Back propagation: The Adam optimizer is selected, and the total loss value ℒ is obtained from expression (19) and backpropagated. After updating the parameter set θ to be trained, step (2) is performed.
(4) Test model: The target domain test set data are inputted into the model, go through the encoder feature extraction module and the fully connected classification module, and the final output label is the diagnosis result of the target domain data.

**Figure 6:**
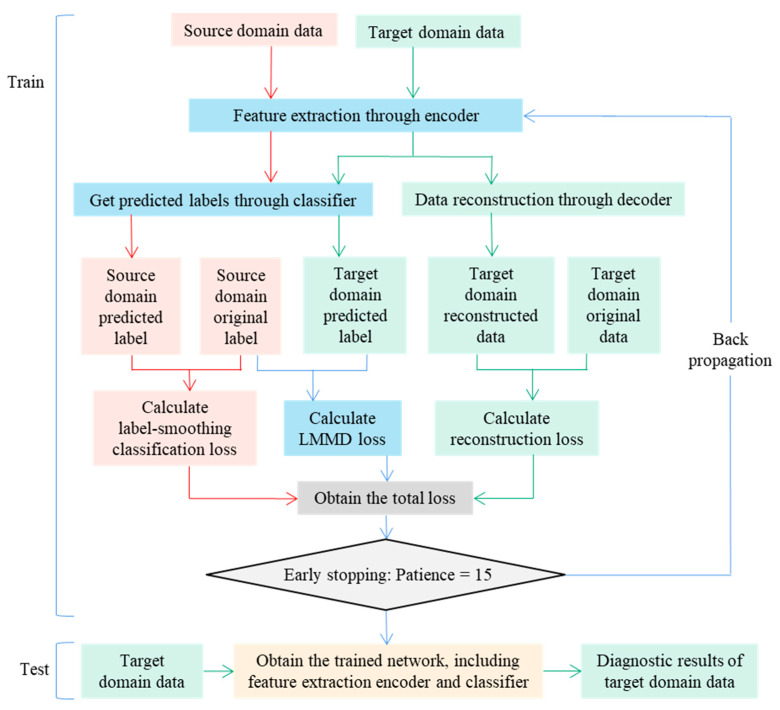
Training and testing flow chart of DRTCNN model.

To sum up, the training and testing process of the DRTCNN is shown in Figure 6. The model execution framework is PyTorch, the optimizer is Adam, the learning rate is 0.001, and the batch size is 64 samples. We use the early stopping mechanism as an abort strategy for model training. The patience is set to 15; that is, training stops when the loss value does not decrease for 15 epochs.

## 4. Experimental Verification of Rolling Bearing Fault Diagnosis

### 4.1. Introduction of Fault Dataset

We evaluated the transfer learning ability of the DRTCNN on three datasets, including the Case Western Reserve University (CWRU) bearing dataset, Beijing Jiaotong University (BJTU) bearing dataset, and Southeast University (SEU) bearing dataset.

Case Western Reserve University bearing dataset: At present, many researchers use the CWRU bearing dataset for fault diagnosis. In this experiment, the drive dataset of CWRU is used, which consists of four different loads: 0 horsepower (HP), 1 HP, 2HP, and 3 HP. It also consists of four different rotational speeds: 1797 r/min, 1772 r/min, 1750 r/min, and 1730 r/min. In order to facilitate the experiment, the datasets A_1_, A_2_, A_3_, and A_4_ are stored, respectively, and fault diagnosis transfer tasks under 12 different operating conditions can be constructed.

Bearings under each working condition have three different fault types: inner race fault (IF), outer race fault (OF), and ball fault (BF). The inner race of the bearing usually fits tightly with the shaft and rotates with the shaft. If fatigue cracks occur, the balls may fall out of the bearings, causing serious machine damage. The bearing outer race cooperates with the bearing seat hole or the mechanical component shell to play a supporting role. If a crack failure occurs, the outer race support force will be insufficient and further deformation will occur, which will affect the bearing’s load-bearing capacity. The rolling elements are evenly arranged between the inner ring and the outer ring. Its damage will directly affect the friction resistance of the bearing during rotation and hinder the smooth operation of the machine. Therefore, the above fault types need to be detected in time to avoid safety accidents.

**Figure 7:**
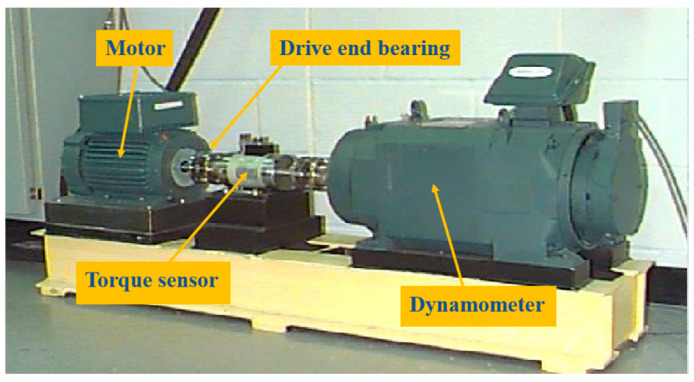
Experimental platform of CWRU bearing dataset.

The bearing dataset provided by the Western Reserve University further classifies the bearing status in more detail according to the fault diameter of the bearing, as shown in Table 3. Each fault category contains three fault diameters of 0.007 inches, 0.014 inches, and 0.021 inches: a total of ten bearing health conditions. The details of the dataset are shown in Table 4. For the data under each working condition, the total number of samples selected is 1000. Of these, 700 samples are selected for training datasets, and the remaining samples are used for testing datasets. The experimental platform for obtaining the CWRU bearing dataset is shown in Figure 7.

**Figure 8:**
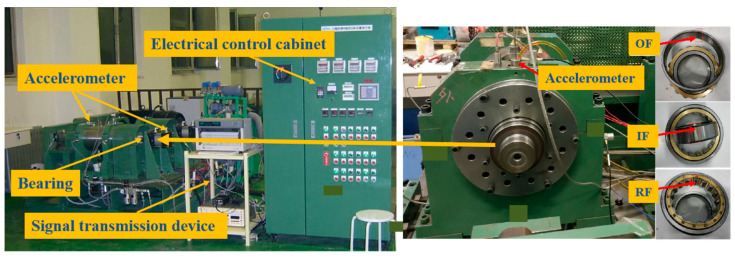
Experimental platform of BJTU bearing dataset.

Beijing Jiaotong University bearing dataset: The vibration signals of the BJTU dataset used in the experiment are collected from three different operating conditions and are related to the bearing speed and load. The details are shown in Table 1. The datasets under five working conditions are stored as B_1_, B_2_, and B_3_, respectively, in order to show that a total of six kinds of cross-domain fault diagnosis transfer tasks can be constructed. The bearings under each condition contain four different health conditions: normal, inner ring failure, outer ring fault, and rolling body failure. For the data of each health condition under each working condition, we selected 1000 samples, of which 300 samples were used for training, and 700 samples were used for testing. The experimental platform for obtaining the BJTU bearing dataset is shown in Figure 8.

**Figure 9:**
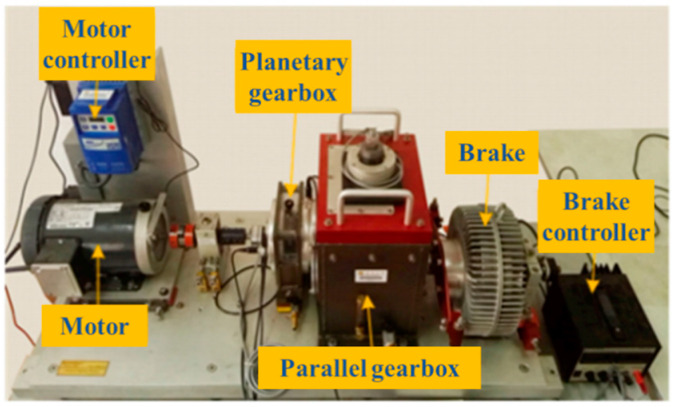
Experimental platform of SEU bearing dataset.

Southeast University bearing dataset: This dataset contains two different speed–load configurations, 20 Hz-0 V and 30 Hz-2 V, which are stored as datasets C_1_ and C_2_, respectively, to facilitate the experiment, and two fault diagnosis transfer tasks under different working conditions can be constructed. The bearings under each working condition have four different health conditions: normal, inner ring failure, outer ring fault, and rolling body failure. The dataset details are shown in Table 1. For the data of each health condition under each working condition, we selected 1000 samples, of which 300 samples were used for training, and 700 samples were used for testing. The experimental platform for obtaining the SEU bearing dataset is shown in Figure 9.

### 4.2. Comparative Study and Design of Models

In order to prove the superiority of the proposed DRTCNN, we choose several newly proposed transfer learning methods and use their feature extraction network and domain alignment strategies to compare experiments. The details of the network structure used are as follows:

(1) DCTLN: the conditional recognition network is constructed using a six-layer CNN, and the MMD algorithm is used to maximize the domain recognition error and minimize the probability distribution distance to complete the transfer.
(2) FTNN: the transferable features are extracted using a two-layer, small convolution kernel CNN, and the domain adaptation is realized by multi-layer domain adaptation and pseudo-label learning through an MMD algorithm.
(3) DASAN: the feature extractor is constructed using a three-layer, small convolution kernel CNN, and the global domain adaptation and subdomain adaptation are realized by combining the LMMD algorithm.
(4) DNCNN: two layers of the CNN are used as the feature extraction layer, and the size of the convolution kernel is 15.
(5) DAHAN: two layers of a wide convolution kernel CNN are used to extract signal features; the first layer uses a wide convolution kernel with a size of 128, and the second layer uses a slightly smaller wide convolution kernel with a size of 64.
(6) MWDAN: the feature extraction part consists of four layers of a CNN: the first layer is wide convolution, and the convolution kernel size is 64; the other convolution layers use a small convolution kernel, where the size is [16, 5, 5].

### 4.3. Experimental Results and Discussion

#### 4.3.1. Analysis of Experimental Results of Fault Diagnosis across Different Working Conditions

In this section, we conducted five experiments on each transfer task for each model and took the average accuracy to evaluate the diagnostic effect of each method.

**Figure 10:**
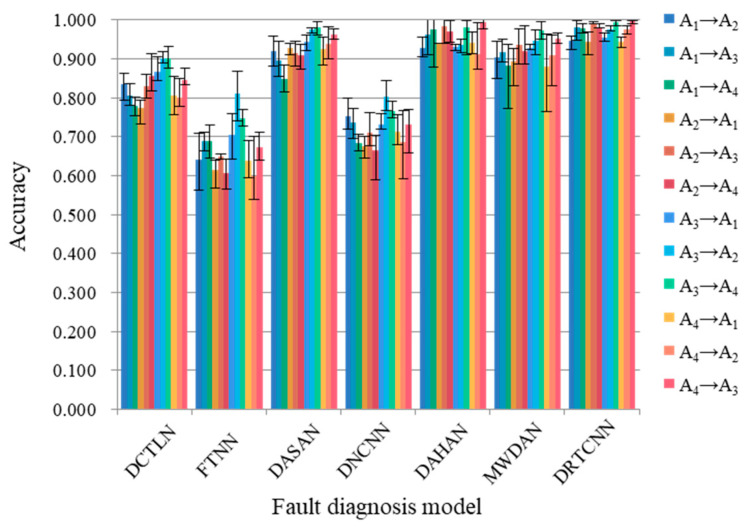
Column chart of diagnostic results for CWRU dataset.

Table 5 shows the experimental results obtained with the CWRU dataset. In order to more directly see the stability of the diagnostic model, we show the maximum and minimum values of the accuracy obtained from five experiments in the column chart, as shown in Figure 10. It can be seen that compared with other methods, the DRTCNN proposed in this paper has a higher fault diagnosis accuracy, and the fluctuation range of the five experimental results is the smallest and has a higher stability. Next, we will compare the DRTCNN with the other six domain adaptive networks from the perspectives of feature extraction and domain transfer, and analyze the reasons why the proposed method achieves a higher accuracy.

From the point of view of feature extraction, the feature extraction part of the FTNN, DNCNN, and DAHAN is only composed of two layers of a CNN, and the network structure is shallow, so the feature extraction ability is poor. In contrast, the DRTCNN uses a deeper convolutional neural network, which can extract deeper transferable features. The feature extractor proposed in this paper is similar to the feature extractor used by the MWDAN and DCTLN, which uses wide kernel convolution in the first layer, which can better extract low-frequency information and filter out the high-frequency noise of the signal, and then extract detailed features through a number of small convolution kernels. Because the main structure of a DRTCNN is an automatic encoder, it is necessary to take into account the feature extraction effect of the encoder and the signal reconstruction effect of the decoder. If the network structure of the encoder is too shallow, it will reduce its feature extraction ability; if the network structure of the decoder is too deep, it will increase the signal reconstruction error, and the difficulty of restoring the signal in the target domain will also increase. Considering that the structure of the encoder and decoder is mirror symmetrical, the DRTCNN uses a five-layer CNN as the encoder, which can well balance the two problems mentioned above, so that the network can not only extract deep transferable features, but also better reconstruct the signal in the target domain, so as to obtain a higher transfer accuracy.

From the perspective of domain transfer, the MMD algorithm used by the DCTLN and FTNN only focuses on global domain adaptation and ignores the differences in subdomain distribution. The LMMD algorithm used by the DRTCNN can take into account the global distribution and subdomain distribution of both the source and target domains, and further align the subdomain distribution by increasing the distance between the classes and reducing the distance within the classes, so it has a better classification effect. The DASAN also uses an LMMD algorithm to achieve a subdomain alignment, but the DRTCNN uses a different LMMD loss weight coefficient, which is defined as:

(21) $$\varepsilon =\frac{2}{1+\exp(-l\cdot s)}-1$$

The definition of the trade-off coefficient used in this model is detailed in Expression (17). The experimental results show that the trade-off coefficient used in this paper is better than that used by the DASAN.

**Figure 11:**
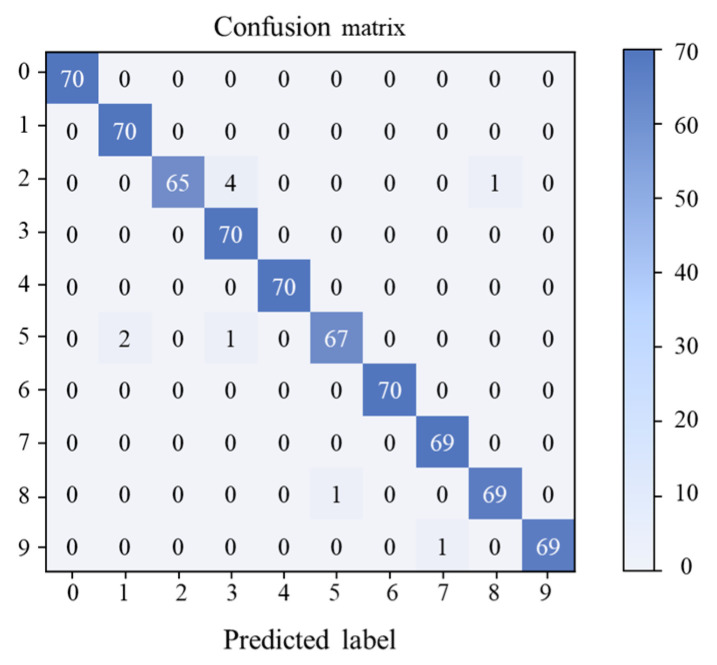
Multi-class confusion matrix of the presented method for CWRU dataset.

In order to further explore the diagnostic accuracy of the DRTCNN model to the test set signal, we use a multi-class confusion matrix, which can show the model’s decision effect on the data more clearly, and the diagnosis result of the transfer task is shown in Figure 11. In Figure 11, the fault categories represented by labels 0 to 9 are as follows: normal, BF007, BF014, BF021, IRF007, IRF014, IRF021, ORF007, ORF014, and ORF021. Specific information on the above nine bearing fault conditions is listed in Table 3. Five samples of label 3 were misclassified, three samples of label 5 were misclassified, only one sample each of label 8 and 9 was misclassified, and the classification accuracies of other fault states reached 100%. The confusion matrix shows the high accuracy diagnosis ability of the model.

**Figure 12:**
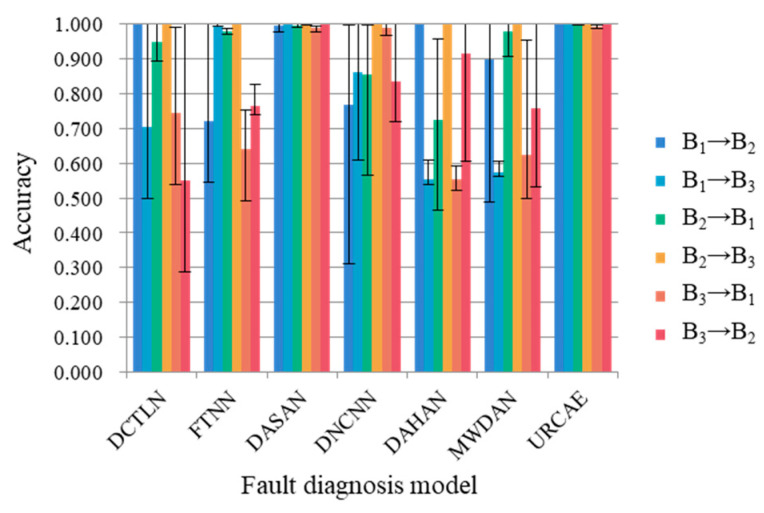
Column chart of diagnostic results for BJTU dataset.

**Figure 13:**
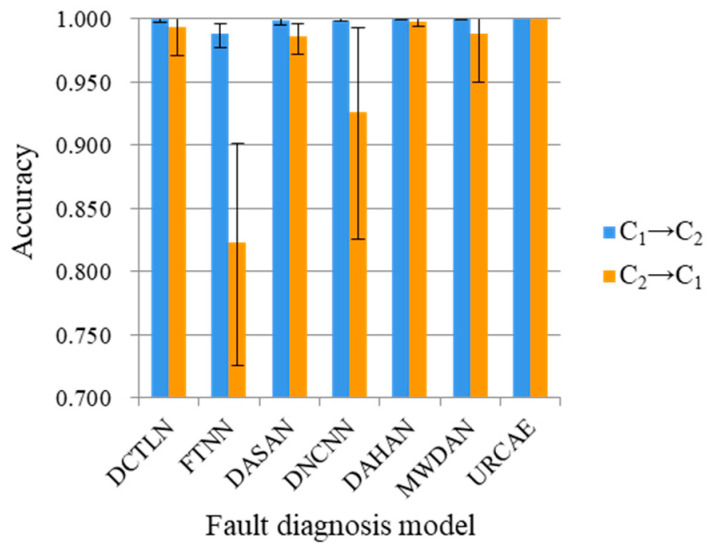
Column chart of diagnostic results for SEU dataset.

In order to prove that the proposed DRTCNN has a high robustness and high generalization ability, we carry out experiments on the BJTU dataset and SEU dataset. Five experiments are carried out for each transfer task, and their average accuracy is calculated, respectively. The diagnostic results are shown in Table 6 and Table 7, and the corresponding bar charts are shown in Figure 12 and Figure 13.

The maximum values of the five experimental results are marked in the graph to show the stability of the model. From the results, it can be seen that the diagnostic accuracy of the model proposed in this paper is higher than that of the six other models, and the numerical fluctuation range in the accuracy of many experiments is small, which shows that the diagnostic effect is stable and has a good robustness. It can achieve better transfer results than the other models on the three datasets from CWRU, BJTU, and SEU at the same time, which shows that the DRTCNN has a better accuracy and stability of fault diagnosis for transfer tasks across different working conditions.

**Figure 14:**
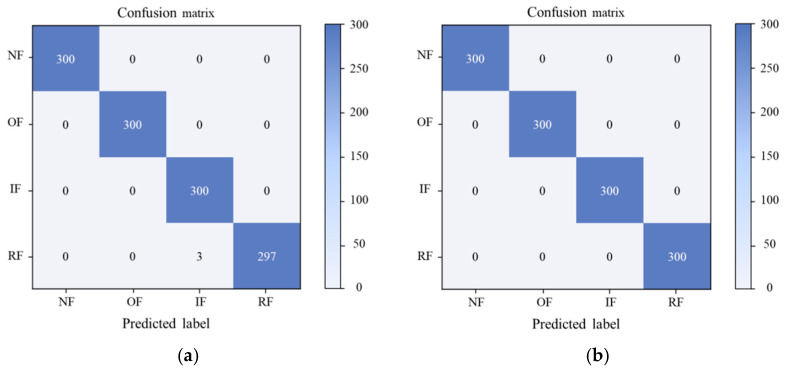
Multi-class confusion matrix for BJTU dataset and SEU dataset. (**a**) BJTU dataset. (**b**) SEU dataset.

As can be seen from the results, the DRTCNN can also achieve better transfer results than the other models on the two other datasets and has a better accuracy and stability. In order to show the classification effect of the model more clearly, taking the “A transition B” transfer task as an example, the multi-class confusion matrix of the experimental diagnosis results is drawn, as shown in Figure 14. In the figure, the fault categories represented by the labels NF, OF, IF, and RF are as follows: normal, inner ring fault, outer ring fault, and roller fault, respectively. As can be seen from Figure 14a, only three samples from label RF are misclassified, and the classification accuracies of the remaining fault states are 100%. In Figure 14b, the classification accuracy of all fault states is 100%. It shows the high accuracy diagnosis ability of the DRTCNN model on the BJTU and SEU datasets.

#### 4.3.2. Ablation Experiment and Results Analysis

In order to ensure that the different components of the DRTCNN have a positive impact on the results of fault diagnosis, we designed an ablation experiment. We record the four ablation models as model M1, model M2, model M3, and model M4, respectively. The different ablation models are as follows:

(1) M1: the local maximum mean difference module is deleted, the subdomains of the source domain and the target domain are no longer aligned in the training process, and the returned loss function only has two parts: classification loss and reconstruction loss.
(2) M2: the decoder module is deleted; that is, the target domain signal is no longer reconstructed, and the loss function consists of two parts: classification loss and LMMD loss; the training of model parameters is only related to data classification and subdomain alignment.
(3) M3: the local maximum mean difference module and the decoder module are deleted at the same time, and the returned loss value is only the classification loss.
(4) M4: the smooth label processing is deleted, the basic cross-entropy loss is used as the classification loss function, and the LMMD loss and reconstruction loss are retained.

We have completed ablation experiments using the data from the CWRU, BJTU, and SEU bearing datasets. First of all, the fault diagnosis accuracy of the experiments on the CWRU bearing dataset is shown in Table 8. It can be seen that after removing the subdomain alignment module, the diagnosis accuracy of the model under different transfer tasks decreases to a certain extent, which proves that the introduction of LMMD loss plays a positive role in the fault diagnosis results and improves the accuracy of the model by 7.43%. After removing the decoder module, the average diagnostic accuracy of the model is reduced by 0.88%, indicating that when the reconstruction module is no longer added to the training process, the effective structure information of the target domain data is not fully utilized, so the reconstruction of the target domain data is necessary. At the same time, after deleting the subdomain alignment module and the decoder module, the diagnosis accuracy of the model decreases more significantly, and the average accuracy decreases by 8.51%, indicating that the combined effect of the two effectively improved the decision accuracy of the network. After using the ordinary cross-entropy loss as the classification loss function, the fault diagnosis accuracy of the model decreases, and overfitting occurs in some transfer tasks, indicating that the smooth label algorithm can effectively restrain the occurrence of overfitting and improve the generalization ability of the diagnosis model.

Similarly, we performed ablation experiments on the BJTU bearing dataset and SEU bearing dataset, and we calculated the average diagnostic accuracy of all cross-domain transfer tasks in five experiments, as well as the average of all accuracy results for each ablation task.

The experimental results using the BJTU dataset and SEU dataset are shown in Table 9 and Table 10, respectively. According to the experimental results, it is not difficult to see that removing the subdomain alignment module reduces the diagnostic accuracy of the model on the BJTU dataset and SEU dataset to a certain extent. This well proves the effectiveness of the subdomain alignment algorithm, which constantly optimizes the distance between the subdomain data based on categories, reduces the distance between similar data, reduces the difficulty of classification, and finally, improves the average accuracy by 26.00% on the BJTU dataset and 13.59% on SEU dataset. When the model removes the decoder, the process of reconstruction of the target domain data is lost, and the average diagnostic accuracies of the model for the BJTU dataset and SEU dataset are reduced by 25.25% and 13.46%, respectively, which confirms the necessity of reconstructing target domain data with autoencoders. When there is only one classifier left in the model, the loss function contains only smooth label cross-entropy loss, and there are only two steps in the model training process: feature extraction and classifier training. Finally, the average accuracy using the BJTU dataset is reduced by 28.90%, and the accuracy using the SEU dataset is reduced by 14.63%. This proves that subdomain alignment and target domain data reconstruction play a significant role in optimizing the fault diagnosis model. The diagnosis accuracy of the cross-domain transfer task is improved. Finally, when the traditional cross-entropy loss is used as the classification loss function, because the smooth label algorithm is no longer used, the fault diagnosis accuracies of some transfer tasks decrease due to over-training fitting, which decreases by 8.14% on the BJTU dataset and 13.67% on the SEU dataset, which shows that the label smoothing algorithm is effective in reducing overfitting and improving the generalization ability of the diagnosis model.

Through the ablation experiments above, we prove that signal reconstruction module, subdomain alignment module, and smooth label processing all have positive effects on the diagnostic accuracy of the DRTCNN. In addition, different modules also promote each other, and they are all indispensable components of the DRTCNN.

#### 4.3.3. Comparative Analysis of Network Structure and Key Parameters

**Figure 15:**
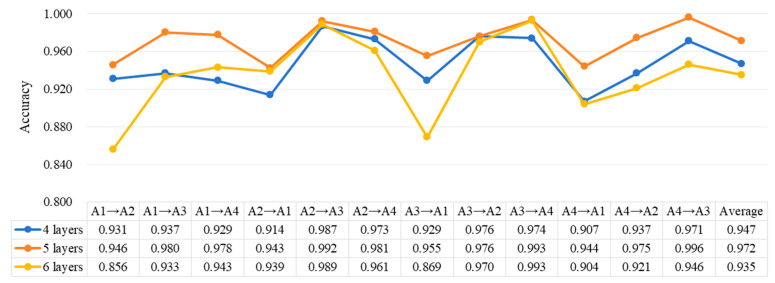
Comparison of the effects of networks with different depths on the CWRU dataset.

The proposed DRTCNN in this article has a five-layer convolutional network. In order to prove that it has a better performance, we use a deeper convolutional network and a shallower convolutional network structure to perform fault diagnosis tasks, respectively, and compare the diagnosis results with the proposed five-layer convolutional network. Based on the proposed network, we added a layer of convolutional block with a convolutional kernel size of 3 × 1 and a pooling kernel size of 2 × 1 to form a deeper six-layer convolutional network. In addition, we constructed a shallower four-layer convolutional network, that is, removing one layer of the convolutional block with a convolutional kernel size of 3 × 1 and a pooling kernel size of 2 × 1. The diagnostic results of the three networks on the CWRU dataset are shown in the Figure 15. It can be seen that the five-layer convolutional neural network has the best diagnostic effect. In general, the receptive field of shallow networks is relatively small, and the fitting effect of the parameters is also limited. The deeper the convolutional layer, the better the feature extraction effect of the network. However, neural networks that are too deep often suffer from network degradation problems and are accompanied by the risk of overfitting. Therefore, it is of great significance to design an appropriate number of layers for the network.

**Figure 16:**
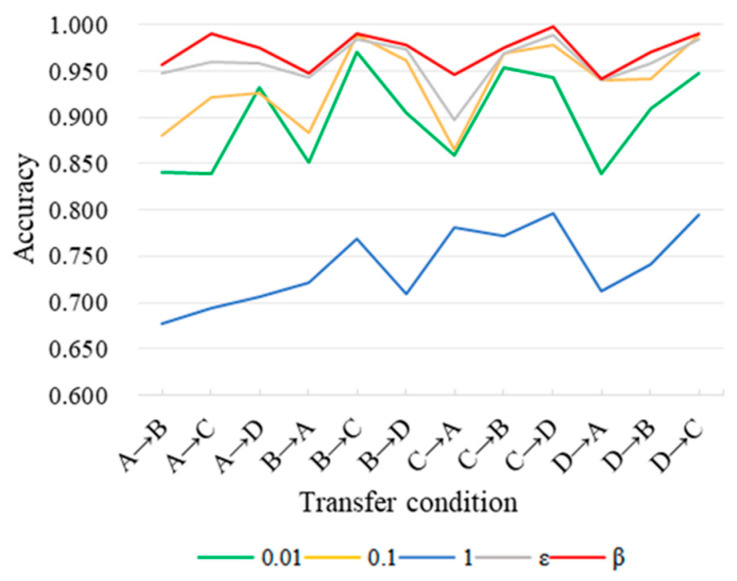
Comparison of the effects of dynamic trade-off factors on the CWRU dataset.

In the method proposed in this paper, the loss function uses a non-fixed domain adaptive coefficient *β*. According to Expression (17), with the increase in training epochs, the value of *β* will transition from 0 to 1, in order to gradually activate LMMD loss and reduce the noise influence in the initial stage of training. In order to verify the validity of the coefficient in this model, we set its values to 0.01, 0.1, and 1, respectively, carried out experiments on the CWRU dataset, and compared the effect of another commonly used adaptive coefficient *ε* in this model. *ε* is defined as Expression (21), and the accuracy of the diagnosis results is shown in Figure 16.

From the comparative experimental results, we can see that when the training parameters take a fixed value, the diagnostic effect of the model is poor. Compared with other commonly used adaptive parameters, the adaptive parameter β used in this model has a better training effect, and the diagnostic model has a higher diagnostic accuracy.

## 5. Conclusions

In order to solve the problem of data distribution drift in rolling bearings caused by a change in the mechanical equipment working conditions, a deep reconstruction transfer convolutional neural network is proposed for fault diagnosis across different working conditions. The proposed method can construct a diagnosis model with a high generalization ability through the bearing signals collected under known operating conditions, reduce the impacts of changeable operating conditions of mechanical equipment on the diagnosis effect, and solve the problem of labeled fault data collection difficulty. The main conclusions are as follows:

(1) The hidden features in the original signal are extracted using the deep reconstruction and transfer network, and combined with a new subdomain alignment algorithm to expand the inter-class distance and reduce the intra-class distance. It is helpful to construct the generalized decision boundary, reduce the influence of the change in working conditions on fault mapping, and effectively improve the recognition accuracy of the model in cross-domain fault diagnosis tasks. In the process of signal reconstruction, the decoder makes full use of the structure information of the target domain data and fully mines the domain invariant features.
(2) Through the construction of a five-layer deep convolution self-encoder with a wide kernel in the first layer, the function of adaptive noise reduction is realized, which does not depend on noise reduction preprocessing and has stronger data feature constraint ability.
(3) In the process of model training, the cross-entropy loss of smooth tags considering confidence is introduced to reduce the trust of wrong tags in the training process, restrain the overfitting of the model, and enhance the effectiveness of feature learning of the network.
(4) Through the verification of 20 transfer tasks constructed by three rolling bearing fault datasets, compared with the existing transfer learning network, the proposed DRTCNN achieves a better fault identification accuracy and generalization performance in the target working conditions and noise environment.

## Figures and Tables

**Table 1:** Details of proposed encoder used in the experiments.

Layer Type	Kernel Size	Stride	Kernel Channel Size
Convolution 1	64 × 1	16 × 1	16
Pooling 1	2 × 1	2 × 1	16
Convolution 2	3 × 1	1 × 1	32
Pooling 2	2 × 1	2 × 1	32
Convolution 3	3 × 1	1 × 1	64
Pooling 3	2 × 1	2 × 1	64
Convolution 4	3 × 1	1 × 1	64
Pooling 4	2 × 1	2 × 1	64
Convolution 5	3 × 1	1 × 1	64
Pooling 5	2 × 1	2 × 1	64
Fully connected	128		1
Softmax	Number of classes		1

**Table 2:** Details of proposed decoder used in the experiments.

Layer Type	Kernel Size	Stride	Kernel Channel Size
Deconvolution 1	3 × 1	1 × 1	64
Upsampling 1	2 × 1	2 × 1	64
Deconvolution 2	3 × 1	1 × 1	64
Upsampling 2	2 × 1	2 × 1	64
Deconvolution 3	3 × 1	1 × 1	32
Upsampling 3	2 × 1	2 × 1	32
Deconvolution 4	3 × 1	1 × 1	16
Upsampling 4	2 × 1	2 × 1	16
Deconvolution 5	64 × 1	16 × 1	1
Upsampling 5	2 × 1	2 × 1	1

**Table 3:** Parameters of 10 fault types in the CWRU dataset.

Fault Location	Class	Fault Diameter	Fault Depth
Inner Raceway	IRF007	0.007 inches	0.011 inches
	IRF014	0.014 inches	
	IRF021	0.021 inches	
Outer Raceway	ORF007	0.007 inches	
	ORF014	0.014 inches	
	ORF021	0.021 inches	
Ball	BF007	0.007 inches	
	BF014	0.014 inches	
	BF021	0.021 inches	

**Table 4:** Parameter description of the bearing dataset.

Name	Class	Condition
A_1_	BF007, BF014, BF021, IRF007, IRF014, IRF021, ORF007, ORF014, ORF021, normal	1797 r/min, 0 HP
A_2_		1772 r/min, 1 HP
A_3_		1750 r/min, 2 HP
A_4_		1730 r/min, 3 HP
B_1_	Normal, ball, inner, outer	2765 RPM, 150 km/h
B_2_		4400 RPM, 250 km/h
B_3_		5270 RPM, 300 km/h
C_1_	Normal, ball, inner, outer	20 Hz, 0 V
C_2_		30 Hz, 2 V

**Table 5:** Recognition results of CWRU dataset.

Method	DCTLN	FTNN	DASAN	DNCNN	DAHAN	MWDAN	DRTCNN
A_1_→A_2_	0.835	0.640	0.921	0.753	0.926	0.904	0.946
A_1_→A_3_	0.805	0.687	0.897	0.737	0.963	0.916	0.980
A_1_→A_4_	0.779	0.688	0.848	0.683	0.976	0.883	0.978
A_2_→A_1_	0.773	0.614	0.927	0.675	0.939	0.894	0.943
A_2_→A_3_	0.830	0.648	0.914	0.711	0.983	0.934	0.992
A_2_→A_4_	0.857	0.605	0.908	0.664	0.971	0.921	0.981
A_3_→A_1_	0.867	0.704	0.943	0.730	0.929	0.927	0.955
A_3_→A_2_	0.901	0.810	0.975	0.803	0.934	0.945	0.976
A_3_→A_4_	0.902	0.747	0.981	0.765	0.980	0.973	0.993
A_4_→A_1_	0.806	0.637	0.924	0.712	0.941	0.879	0.944
A_4_→A_2_	0.800	0.599	0.939	0.687	0.912	0.910	0.975
A_4_→A_3_	0.845	0.672	0.963	0.732	0.995	0.951	0.996

**Table 6:** Recognition results of BJTU dataset.

Method	DCTLN	FTNN	DASAN	DNCNN	DAHAN	MWDAN	DRTCNN
B_1_→B_2_	1.000	0.722	0.995	0.770	1.000	0.898	1.000
B_1_→B_3_	0.706	0.998	1.000	0.861	0.555	0.575	1.000
B_2_→B_1_	0.949	0.978	0.997	0.857	0.726	0.980	0.999
B_2_→B_3_	1.000	1.000	0.999	1.000	1.000	1.000	1.000
B_3_→B_1_	0.747	0.641	0.989	0.990	0.554	0.623	0.993
B_3_→B_2_	0.550	0.764	1.000	0.835	0.916	0.760	1.000

**Table 7:** Recognition results of SEU dataset.

Method	DCTLN	FTNN	DASAN	DNCNN	DAHAN	MWDAN	DRTCNN
C_1_→C_2_	1.000	0.989	0.999	0.999	1.000	1.000	1.000
C_2_→C_1_	0.994	0.823	0.986	0.926	0.998	0.989	1.000

**Table 8:** Ablation study of CWRU dataset.

Method	M_1_	M_2_	M_3_	M_4_	DRTCNN
A_1_→A_2_	0.864	0.950	0.847	0.957	0.960
A_1_→A_3_	0.853	0.976	0.836	0.944	0.990
A_1_→A_4_	0.833	0.971	0.829	0.943	0.976
A_2_→A_1_	0.861	0.944	0.857	0.920	0.949
A_2_→A_3_	0.977	0.989	0.974	0.986	0.990
A_2_→A_4_	0.910	0.969	0.904	0.970	0.979
A_3_→A_1_	0.867	0.917	0.853	0.920	0.946
A_3_→A_2_	0.963	0.969	0.947	0.957	0.976
A_3_→A_4_	0.956	0.997	0.951	0.981	0.999
A_4_→A_1_	0.846	0.927	0.824	0.923	0.941
A_4_→A_2_	0.903	0.976	0.897	0.930	0.971
A_4_→A_3_	0.939	0.986	0.921	0.976	0.990
Avg.	0.898	0.964	0.887	0.951	0.972

**Table 9:** Ablation study of BJTU dataset.

Method	M_1_	M_2_	M_3_	M_4_	DRTCNN
B_1_→B_2_	0.708	1.000	0.697	1.000	1.000
B_1_→B_3_	1.000	0.508	0.890	0.508	1.000
B_2_→B_1_	0.764	1.000	0.644	1.000	1.000
B_2_→B_3_	1.000	1.000	0.782	1.000	1.000
B_3_→B_1_	0.750	0.982	0.513	0.994	0.998
B_3_→B_2_	0.728	1.000	0.725	0.995	1.000
Avg.	0.825	0.915	0.708	0.916	1.000

**Table 10:** Ablation study of SEU dataset.

Method	M_1_	M_2_	M_3_	M_4_	DRTCNN
C_1_→C_2_	0.981	0.736	0.976	0.738	1.000
C_2_→C_1_	0.747	0.994	0.731	0.988	0.999
Avg.	0.864	0.865	0.853	0.863	1.000

## Data Availability

Data are contained within the article.
